# Grief and Economic Stressors by Sex, Gender, and Education: Associations With Alzheimer Disease–Related Outcomes

**DOI:** 10.1212/WNL.0000000000213377

**Published:** 2025-03-31

**Authors:** Eleni Palpatzis, Muge Akinci, Marina Garcia-Prat, Kaj Blennow, Henrik Zetterberg, Clara Quijano-Rubio, Gwendlyn Kollmorgen, Norbert Wild, Juan Domingo Gispert, Marc Suárez-Calvet, Oriol Grau-Rivera, Karine Fauria, Anna Brugulat-Serrat, Gonzalo Sanchez-Benavides, Eider M. Arenaza-Urquijo, for the ALFA study

**Affiliations:** 1 Barcelona Institute for Global Health (ISGlobal), Spain;; 2 University of Pompeu Fabra (UPF), Spain;; 3 Barcelonabeta Brain Research Center, Spain;; 4 Department of Psychiatry and Neurochemistry, University of Gothenburg, Sweden;; 5 Clinical Neurochemistry Laboratory, Sahlgrenska University Hospital, Sweden;; 6 UK Dementia Research Institute, University College London, United Kingdom;; 7 Department of Neurodegenerative Disease, University College London, United Kingdom;; 8 Hong Kong Center for Neurodegenerative Diseases, China;; 9 Wisconsin Alzheimer's Disease Research Center, University of Wisconsin School of Medicine and Public Health, Madison;; 10 Roche Diagnostics International Ltd., Switzerland;; 11 Roche Diagnostics GmbH, Germany;; 12 Hospital del Mar Medical Research Institute, Spain;; 13 Centro de Investigación Biomédica en Red de Fragilidad y Envejecimiento Saludable (CIBERFES), Spain;; 14 Servei de Neurologia, Hospital del Mar, Spain;; 15 Centro de Investigación Biomédica en Red Bioingeniería, Instituto de Salud Carlos III, Spain; and; 16 Global Brain Health Institute, University of California, San Francisco.

## Abstract

**Background and Objectives:**

The prevalence and impact of stressful life events (SLEs) on age-related and Alzheimer disease (AD)–related pathways may depend on social determinants including gender and education. We investigated whether specific SLEs are associated with AD pathology and neurodegeneration and how these associations differ by gender and education.

**Methods:**

This cross-sectional study included cognitively unimpaired participants, most with a family history of sporadic AD, from the ALzheimer's and FAmilies (ALFA) cohort, based in Barcelona, Spain. Participants had available assessments on occurrence and type of lifetime SLEs and lumbar puncture and/or structural MRI. We performed multiple regression analyses to examine the associations of specific SLE type with (1) AD pathologies (CSF phosphorylated tau 181 [p-tau181] and β-amyloid [Aβ] 42/40) and (2) neurodegeneration markers (CSF neurogranin and GM volumes voxel-wise) including interaction and stratification analyses by gender (women/men) and education.

**Results:**

In total, 1,290 cognitively unimpaired participants (mean age = 59.4 years, range: 48–77 years, 99% White participants, 61% women) were included (393 with lumbar puncture and 1,234 with spectroscopic MRI assessments). Less educated participants and women reported more grief-related and economic-related SLEs. Furthermore, women reported more abuse and reproductive SLEs. Grief-related SLEs were associated with AD and neurodegeneration CSF outcomes while economic SLEs were associated with MRI-based GM outcomes, both in an age-independent manner. Specifically, partner's death was associated with lower Aβ42/40 (B = −5.19; 95% CI −9.61 to −0.76; *p* = 0.022) and higher p-tau181 (B = 0.18; 95% CI 0.05–0.32; *p* = 0.007) and neurogranin (B = 0.19; 95% CI 0.05–0.32; *p* = 0.007). The associations with Aβ42/40 were driven by less educated participants and men and associations with p-tau181 and neurogranin driven by women. Unemployment and economic loss were associated with lower GM volumes in limbic and frontal areas, driven by more educated participants and men and by women, respectively.

**Discussion:**

Older adults at risk of cognitive decline with less education and women may be more susceptible to experience more SLEs. Men who have experienced widowhood and unemployment and women who have experienced financial difficulties may benefit from interventions.

## Introduction

A largely unexplored potential risk factor of Alzheimer disease (AD) and dementia is the lifetime exposure to stressful life events (SLEs). SLEs are objective and observable major events that typically require life readjustment^1^ and perceived as threats to an individual's well-being, identity, or social status.^2^ Our previous work suggests a mechanistic link between increased exposure to SLEs and higher AD pathophysiologic markers later in life, influenced by history of psychiatric disease and gender.^3^

Previous research has suggested that SLEs play a significant role in shaping social disparities among racially and ethnically minoritized groups.^4^ It is well established that AD prevalence is higher among women and less educated individuals,^5,6^ groups that also experience a higher prevalence of SLEs.^7,8^ The prevalence and impact of specific SLEs may also differ within groups, as, for example, unemployment is more common among individuals with less education.^9^ However, the impact of specific SLEs on AD remains understudied, with few existing studies focusing on isolated SLEs rather than on multiple SLEs. These studies have shown associations of losing a parent^10^ and having worse socioeconomic circumstances in childhood^11^ or getting divorced^12^ with an increased AD risk. Widowhood was associated with accelerated β-amyloid (Aβ)–related cognitive decline among cognitively unimpaired older men and women.^13^

Therefore, we aim to examine the association of specific SLEs with AD pathophysiology and brain structure and assess the effect of gender and education among cognitively unimpaired individuals at risk of cognitive decline. We hypothesized to observe a higher prevalence of SLEs among women and those less educated and a differential impact of SLEs on brain health depending on gender and educational level.

## Methods

### Study Design, Setting, and Participants

This cross-sectional cohort study consists of participants of the ALFA (for ALzheimer's and FAmilies) parent cohort that includes 2,743 cognitively unimpaired participants at an increased risk of AD (86% have at least 1 parent diagnosed with AD^14^) who were enrolled between 2013 and 2014 (eFigure 1). Participants were recruited through press recruitment (eTable 1 provides inclusion and exclusion criteria).^14^

A subset (n = 419) of the ALFA parent cohort participants are part of the nested longitudinal ALFA+ study invited based on their enriched AD risk profile (AD parental history, *APOE* ε4 status, verbal episodic memory score, and the Cardiovascular Risk Factors, Aging, and Incidence of Dementia risk score).^14^ The ALFA+ participants underwent advanced protocols of structural MRI, amyloid PET, and lumbar puncture for CSF biomarker assessments.

In this study, we included all participants from the ALFA+ cohort with complete-case data available on CSF measurements and across all other variables (AD pathophysiology sample) and all participants from the ALFA parent cohort with complete-case data available on MRI-based gray matter volume and across all other variables (brain structure MRI sample). eFigure 1 depicts the flow diagram of participant selection for this study. This study followed the Strengthening the Reporting of Observational Studies in Epidemiology reporting guideline.

### Standard Protocol Approvals, Registrations, and Patient Consents

The ALFA and ALFA+ study protocols have been approved by the Independent Ethics Committee “Parc de Salut Mar,” Barcelona, and registered at ClinicalTrials.gov (identifiers: NCT01835717, NCT02485730). Participants provided informed consent at baseline with a form approved by the Independent Ethics Committee “Parc de Salut Mar,” Barcelona. All methods were performed in accordance with the ethical standards as laid down in the 1964 Declaration of Helsinki and its later amendments or comparable ethical standards.

### Demographic and Other Characteristics

Demographic and lifestyle characteristics used in this study were collected at participants' latest study visit between 2016 and 2021 through interviews and self-reported questionnaires and included age; gender; years of education as a continuous covariate; and history of cardiovascular disease and psychiatric disease, both as binary covariates. Participants self-reported their race-ethnic self-identification (White, North African, Sub-Saharan African, Latin American, Asian, Roma, and other ethnicities) and their gender using a binary label (i.e., woman/man). Sex refers to an individual's sex chromosome complement (XX vs XY) and is typically assigned at birth based on external anatomy while gender is a social construct referring to socially constructed roles, behaviors, and identities.^15^ Sex and gender are intrinsically linked, but because the aim in our study is to mainly understand the effects of stressors in a gender-oriented framework, in which women and men have, for example, different socially constructed roles and opportunities for work/education, we will, therefore, refer to women/men throughout the article. Conversely, we will refer to female/male when discussing potential biological pathways. Participants were regarded as having a history of cardiovascular or psychiatric disease if they self-reported having received a clinical diagnosis at any point during their lifetime. Participants were also genotyped for 2 single-nucleotide variants, rs429358 and rs7412, which dictate the protein isoform generated from *APOE*. *APOE* ε4-carrier status was included as a binary covariate.

### Specific SLEs

Participants underwent clinical interviews between 2016 and 2021. Exposure to 18 different stressors was examined with a structured interview with predefined events adopted from the SNAC-K study,^16^ based on Miller and Rahe's list of life change events (eTable 2).^1,17^ Assessing SLEs through structured interviews have been found to have better test-retest reliability and reduce intracategory variability compared with traditional SLE checklists.^18^ Indeed, our SLE measurements showed high test-retest correlation.^3^ We ran test-retest correlations to assess the reliability of our measurement in participants with 2 assessments. The SLE measurements showed a high test-retest correlation within the whole CSF (*r* = 0.93, *p* < 0.001) and MRI (*r* = 0.93, *p* = 0.001; mean time in-between assessments = 4.0 and 3.9 years, respectively) samples.^3^

Participants reported whether each event had happened during their lifetime at least once. For participants who underwent the interview more than once, only their last interview was considered. The events were categorized into binary variables based on whether the event occurred at least once (0 = event not experienced, 1 = event experienced once or more). Information on the count of repeated events and timing of events within the sample has been described elsewhere.^3^ For this study, we included SLEs with a prevalence of 5% or more within our samples, resulting in the inclusion of 13 different SLEs (Table 1).

**Table 1:** List of Stressors and the Frequencies of Having Experienced Each SLE Within the CSF (n = 393) and MRI (n = 1,234) Samples

	CSF sample (n = 393)			MRI sample (n = 1,234)		
	Times each event happened in lifetime Mean (SD)	No. of participants having experienced the SLE at least once (%)	Age at time of event^a^ Mean (SD; range; mode)	Times each event happened in lifetime Mean (SD)	No. of participants having experienced the SLE at least once (%)	Age at time of event^a^ Mean (SD; range; mode)
Death of the mother	0.75 (0.44)	293 (74.6)	53.6 (8.0; 24–70; 57)	0.67 (0.47)	823 (66.7)	52.1 (9.4; 0–72; 50)
Death of the father	0.88 (0.33)	344 (87.5)	46.1 (12.5; 3–75; 48)	0.82 (0.38)	1,015 (82.3)	44.6 (12.5; 0–75; 48)
Abuse	0.07 (0.27)	28 (7.1)	9.0 (9.4; 1–25; 10)	0.13 (0.38)	151 (12.2)	17.9 (14.1; 1–61; 10)
Terminated pregnancy	0.31 (0.62)	95 (24.2)	29.7 (6.2; 16–46; 30)	0.31 (0.64)	292 (23.7)	29.7 (6.8; 16–52; 28)
Divorce	0.20 (0.43)	74 (18.8)	40.8 (10.2; 22–64; 40)	0.29 (0.53)	317 (25.7)	39.3 (10.0; 21–69; 40)
Death of partner	0.10 (0.30)	37 (9.4)	51.5 (13.2; 15–70: 61)	0.07 (0.25)	81 (6.6)	47.5 (15.8; 15–73; 50)
Death of a close one	0.48 (0.73)	142 (36.1)	43.1 (17.4; 1–69; 47)	0.56 (0.80)	518 (42.0)	41.6 (15.8; 1–73; 45)
Major illness	0.09 (0.33)	33 (8.4)	44.8 (17.4; 3–68; 57)	0.13 (0.37)	144 (11.7)	41.0 (16.3; 1–69; 57)
Major illness of a close one	0.83 (0.38)	325 (82.7)	41.8 (10.74; 5–66; 50)	0.87 (0.34)	1,073 (87.0)	40.5 (10.9; 1–73; 40)
Becoming unemployed	0.35 (0.58)	121 (30.8)	41.4 (12.3; 18–63; 30)	0.37 (0.57)	405 (32.8)	41.0 (11.9; 18–68; 45)
Retirement	0.42 (0.49)	166 (42.2)	61.5 (4.3; 36–70; 65)	0.31 (0.46)	378 (30.6)	61.6 (3.9; 44–70; 65)
Severe financial loss	0.11 (0.33)	40 (10.2)	43.9 (9.4; 29–61; 35)	0.18 (0.43)	203 (16.5)	42.9 (10.1; 18–68; 40)
Financial issues in family while growing up	0.10 (0.30)	40 (10.2)	10.6 (5.5; 1–23; 10)	0.08 (0.28)	104 (8.4)	10.6 (5.0; 1–23; 10)

Abbreviation: SLE = stressful life event.

a Only the ages at first-ever events were considered.

### CSF Measurements

Participants underwent lumbar puncture between years 2016 and 2019. The average number of days elapsed from lumbar puncture to assessment of SLEs was 511.36 days (SD = 593.78, range = −582 to 1,512 days). CSF phosphorylated tau 181 (p-tau181) was measured using the Elecsys Phospho-Tau (181P) CSF electrochemiluminescence immunoassay on a fully automated cobas e 601 instrument (Roche Diagnostics International Ltd., Rotkreuz, Switzerland). Aβ42/40 ratio and neurogranin were measured with the NeuroToolKit, a panel of robust prototype assays (Roche Diagnostics International Ltd.), on a cobas e 411 or e 601 instrument. All CSF measurements were performed at the Clinical Neurochemistry Laboratory at Sahlgrenska University Hospital, Sweden.^19^

Owing to non-normality of the residuals in the models of Aβ42/40 ratio_,_ p-tau, and neurogranin, these variables were log_10_-transformed.

### MRI Acquisition and Image Preprocessing for Voxel-Based Morphometry

Participants underwent MRI scans between years 2016 and 2019. The average number of days elapsed from MRI scans to assessment of SLEs was 56.04 days (SD = 209.95, range = −371 to 1,573 days). The T1-weighted 3D turbo field-echo sequence was acquired in a Philips (Best, the Netherlands) 3T Ingenia CX scanner with a voxel size of 0.75 × 0.75 × 0.75 mm^3^, field of view of 240 × 240 × 180 mm^3^, sagittal acquisition, flip angle of 8°, repetition time of 9.9 milliseconds, echo time of 4.6 milliseconds, and inversion time of 900 milliseconds. The VBM preprocessing was performed using SPM12 software running on MATLAB R2010a. Images were first segmented into gray matter, white matter, and CSF using an extended set of probability maps. Next, Dartel was used to estimate accurate spatially normalizing transformations, after which the gray matter maps were smoothed by convolving with an isotropic Gaussian kernel of 8-mm full width half maximum. A mean normalized group-level gray matter mask was generated from individual segmented masks and thresholded at gray matter probability >0.3. Cerebellar and vermian areas were removed from the mask.

### Statistical Analyses

Characteristics of the sample were summarized as means and SDs for continuous variables and as frequencies and percentages for categorical variables. We used χ^2^ tests to examine whether the prevalence of SLEs differed by gender and educational level. To examine the association of specific SLEs with the CSF outcomes, we performed 3 sets of multivariable regression analyses adjusting for age, gender, years of education, *APOE* ε4-carrier status, history of cardiovascular and psychiatric diseases, and all other SLEs. Moreover, analyses were stratified by gender and educational level, and interaction analyses were performed. For stratified and interaction analyses, education was categorized into low (12 or fewer years) and high (13 or more years) levels of education.

Because we considered the occurrence of SLEs alone, in sensitivity analyses, to ensure the robustness of our results, we repeated the analyses with CSF variables as outcomes by adjusting for the total accumulated life events (taking into account how many times each SLE was experienced) and we also repeated the analyses by not adjusting for any other SLEs.

*p* Values were 2-sided with statistical significance set at less than 0.05. All analyses were prespecified and performed with Stata SE, version 17.0 (StataCorp., College Station, TX). Given that all analyses were prespecified and we expected low-to-moderate effects of SLEs in a preclinical sample, we did not use correction for multiple comparison methods because they are too conservative and increase type II error.^20^

### Voxel-Wise Statistical Analyses

We used multiple regression in SPM to investigate the association of specific SLEs with gray matter volume including age, gender, years of education, *APOE* ε4 status, history of cardiovascular and psychiatric diseases, total intracranial volume as covariates, and all the specific SLEs in the statistical analyses. All the SLEs were considered. We considered significant results surviving an uncorrected *p* value of 0.001 at voxel level and a cluster-level *p* < 0.05 family-wise error (FWE) correction.

### Data Availability

The data supporting the findings of this study may be available on a reasonable request from the ALFA study management team.

## Results

Characteristics of the participants included in the study are given in Table 2 and characteristics by gender and educational level in eTables 3 and 4. Among the 393 participants included in the CSF sample, the mean (SD) age was 61.2 (4.7) years at lumbar puncture, and among the MRI participants, the mean (SD) age at MRI was 59.2 (6.6) years. There was an overlap of 337 participants in the CSF and MRI samples. The differences in participant characteristics between the ALFA parent cohort and the participants included in this study are provided in eTable 5.

**Table 2:** Characteristics of the CSF Sample (n = 393) and MRI Sample (n = 1,234)

Characteristics	CSF sample (n = 393)	MRI sample (n = 1,234)	All participants (n = 1,290)
Gender (women)	242 (61.6)	764 (61.9)	792 (61.4)
Race-ethnic self-identification (White)^a^	391 (99.5)	1,225 (99.3)	1,281 (99.3)
History of cardiovascular disease (yes)	160 (40.7)	382 (31.0)	408 (31.6)
History of psychiatric disease (yes)	120 (30.5)	374 (30.3)	390 (30.3)
*APOE* ε4-carrier (yes)	210 (53.4)	493 (40.0)	525 (40.7)
At least 1 parent diagnosed with AD dementia (yes)	345 (87.8)	912 (73.9)	947 (73.4)
Self-reported SES^b^			
Low	27 (7.3)	27 (7.3)	27 (7.3)
Medium	306 (82.7)	306 (82.7)	306 (82.7)
High	37 (10.0)	37 (10.0)	37 (10.0)
Age at lumbar puncture (y)	61.2 (4.7; 49.3–73.6)	—	—
Age at MRI (y)	—	59.2 (6.6; 47.6–76.8)	—
Years of education completed	13.5 (3.5; 6–20)	13.6 (3.5; 5–20)	13.6 (3.5; 5–20)
Aβ42/40 ratio × 100^c^	7.5 (2.0; 2.0–14.2)	—	—
Phosphorylated tau_181_ (pg/mL)	16.3 (7.6; 7.9–81.1)	—	—
Neurogranin (pg/mL)	799.0 (331.0; 219.0–2,380.0)	—	—

Abbreviations: Aβ = β-amyloid; SES = socioeconomic status.

Data are presented as n (%) or mean (SD; range).

a Race and ethnic self-identification data were collected with a close-ended question including a mix of both categories as answers (White, North African, Sub-Saharan African, Latin American, Asian, Roma, and other ethnicities).

b Data available for 370 participants within both CSF and brain integrity samples.

c Values of CSF log_10_ Aβ42/40 ratio multiplied by 100.

### Prevalence of SLEs by Education and Gender Groups

**Figure 1:**
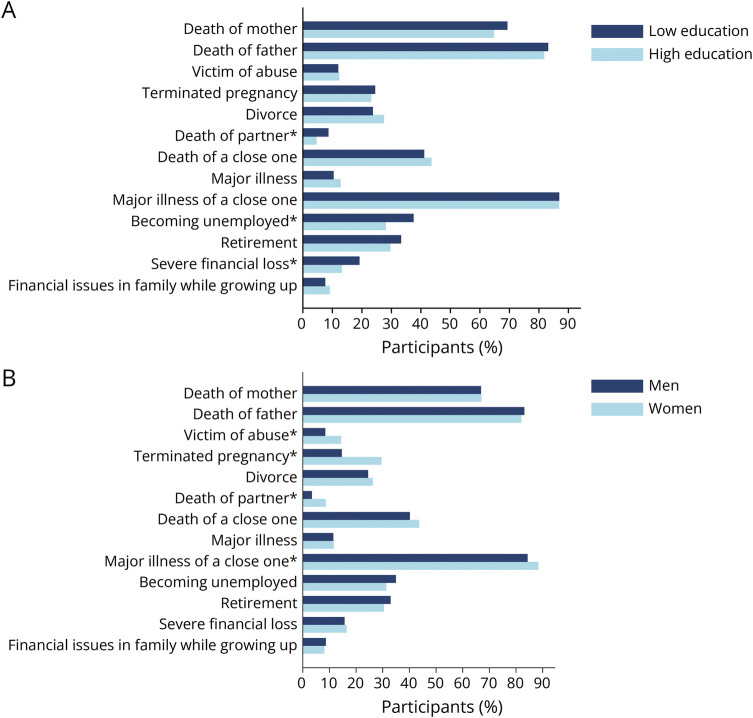
Percentage of Participants Who Have Experienced Each SLE At Least Once in Their Lifetime Within the Whole Sample (n = 1,290) (A) Stratified by education. Asterisks represent significant differences (*p* < 0.05) in prevalence between those with lower and higher educational levels. (B) Stratified by gender. Asterisks represent significant differences (*p* < 0.05) in prevalence between men and women. SLE = stressful life event.

Within the whole study sample (n = 1,290), participants with a lower educational level (≤12 years), compared with those with a higher educational level (≥13 years), reported a higher prevalence of death of partner (8.6% vs 4.6%; *p* = 0.004), unemployment (37.5% vs 28.1%; *p* < 0.001), and severe financial loss (19.1% vs 13.2%; *p* = 0.004; Figure 1A). Within the whole study sample (n = 1,290), women, compared with men, reported a higher prevalence of having been a victim of abuse (14.4% vs 8.4%; *p* = 0.001), terminated pregnancy (within the partnership) (29.6% vs 14.7%; *p* < 0.001), death of partner (*p* < 0.001), and major illness of a close one (88.4% vs 84.3%; *p* = 0.036; Figure 1B). The prevalences are further indicated separately for the 2 samples (CSF sample, n = 393; MRI sample, n = 1,234) in eTables 6 and 7.

### Association of Specific SLEs With Amyloid, Tau, and Neurogranin

Within the whole CSF sample, independent of all covariates and other SLEs, death of partner was associated with a lower Aβ42/40 ratio (*p* = 0.022) and higher p-tau (*p* = 0.007) and neurogranin (*p* = 0.007) levels (Table 3). No other stressors were associated with any of the CSF outcomes.

**Table 3:** Unstandardized Regression Coefficients (95% CIs; *p* Values) for Having Experienced (vs Not) Specific SLEs Across the Life Course in Alzheimer Disease Pathophysiologic Markers (n = 393)

Predictors	Log_10_ Aβ42/40 ratio^a^		Log_10_ phosphorylated tau181		Log_10_ neurogranin	
	Coefficient (95% CI)	*p* Value	Coefficient (95% CI)	*p* Value	Coefficient (95% CI)	*p* Value
Death of the mother	1.06 (−2.00 to 4.12)	0.495	0.08 (−0.01 to 0.17)	0.091	0.08 (−0.01 to 0.17)	0.099
Death of the father	−1.94 (−5.89 to 2.02)	0.337	0.05 (−0.07 to 0.17)	0.375	−0.01 (−0.13 to 0.11)	0.931
Abuse	1.79 (−3.16 to 6.73)	0.477	0.09 (−0.06 to 0.24)	0.221	0.12 (−0.03 to 0.27)	0.109
Terminated pregnancy	−0.69 (−3.66 to 2.28)	0.648	0.06 (−0.03 to 0.15)	0.214	0.07 (−0.02 to 0.16)	0.143
Divorce	−0.16 (−3.55 to 3.23)	0.925	−0.06 (−0.16 to 0.04)	0.256	−0.10 (−0.20 to 0.00)	0.053
Death of partner	−5.19 (−9.61 to −0.76)	0.022	0.18 (0.05 to 0.32)	0.007	0.19 (0.05 to 0.32)	0.007
Death of a close one	−0.62 (−3.28 to 2.04)	0.647	0.04 (−0.04 to 0.12)	0.299	0.05 (−0.03 to 0.13)	0.201
Major illness	1.24 (−3.42 to 5.89)	0.602	0.00 (−1.45 to 0.14)	0.945	−0.05 (−0.19 to 0.09)	0.498
Major illness of a close one	−3.03 (−6.49 to 0.44)	0.087	−0.03 (−0.13 to 0.08)	0.611	−0.05 (−0.15 to 0.06)	0.401
Becoming unemployed	−0.91 (−3.75 to 1.92)	0.527	−0.06 (−0.14 to 0.03)	0.199	−0.07 (−0.16 to 0.02)	0.113
Retirement	−0.15 (−3.58 to 3.28)	0.932	−0.05 (−0.15 to 0.06)	0.359	−0.07 (−0.18 to 0.03)	0.179
Severe financial loss	0.69 (−3.76 to 5.14)	0.761	0.07 (−0.07 to 0.20)	0.333	0.11 (−0.02 to 0.25)	0.095
Financial issues in family while growing up	2.00 (−2.21 to 6.21)	0.351	−0.04 (−0.16 to 0.09)	0.586	0.00 (−0.13 to 0.13)	0.999

Abbreviations: Aβ = β-amyloid; SLE = stressful life event.

Adjusted for gender, age, years of education, *APOE* ε4-carrier status, history of cardiovascular disease, history of psychiatric disease, and all other stressors.

a Values of CSF log_10_ Aβ42/40 ratio multiplied by 100 to facilitate the interpretation of the results.

### Voxel-Wise Association of Specific SLEs With Brain Structure

**Figure 2:**
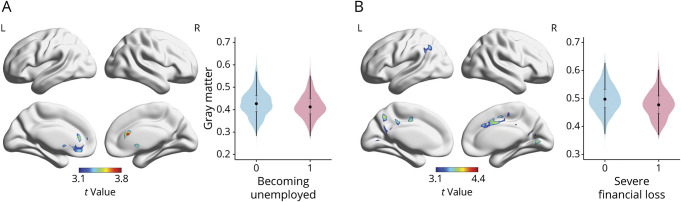
Results of the Voxel-Wise Analyses Showing the Association of Specific SLEs With Reduced Regional Gray Matter Volumes Within the Whole MRI Sample Y-axes represent the probability of gray matter within the significant clusters identified in SPM analyses. X-axes represent having experienced a stressful life event (0 = no, 1 = yes). (A) Unemployment: thresholded at *p* < 0.001 at voxel level and family-wise error *p* < 0.05 at cluster level. (B) Severe financial loss: thresholded at *p* < 0.001 at voxel level and family-wise error *p* < 0.05 at cluster level. All models were adjusted for age, gender, education, total intracranial volume, *APOE* ε4-carrier status, history of cardiovascular disease, history of psychiatric disease, and all other stressful life events. SLE = stressful life event.

Associations of specific SLEs and regional gray matter volumes are shown in Figure 2. Becoming unemployed was associated with reduced gray matter volume in the right and left anterior cingulate gyrus, extending to the left paracingulate gyrus and in the subcallosal cortex (Figure 2A). Severe financial loss was associated with reduced gray matter volume bilaterally in the lingual gyrus, precuneus, precentral gyrus, and anterior cingulate gyrus, extending to the juxtapositional lobule cortex and the paracingulate gyrus, as well as with reduced gray matter volume in the left angular and supramarginal gyrus (Figure 2B). These results were independent of gender, age, *APOE* ε4-carrier status, history of cardiovascular and psychiatric diseases, years of education, and other SLEs. No other specific SLEs were associated with gray matter volumes.

### Association of Specific SLEs With Amyloid, Tau, Neurogranin, and Brain Structure by Gender

In analyses stratified by gender, death of partner was associated with a lower Aβ42/40 ratio (*p* = 0.011; eTable 8) only among men and with higher p-tau (*p* = 0.008; eTable 9) and neurogranin (*p* = 0.006; eTable 10) levels only among women. There was no interaction of death of the partner with gender on Aβ, p-tau, or neurogranin (eTable 11).

**Figure 3:**
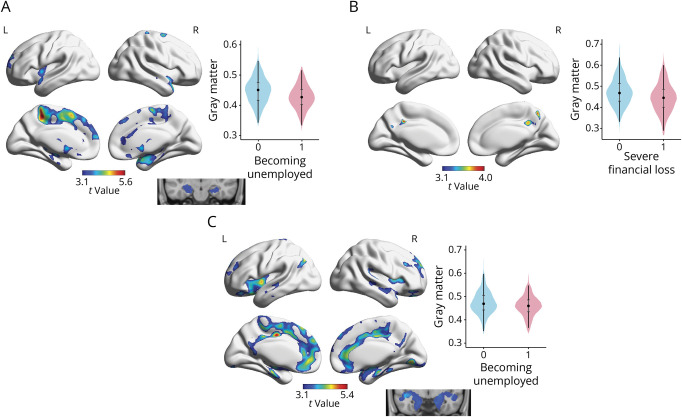
Results of the Voxel-Wise Analyses Showing the Association of Specific SLEs With Reduced Regional Gray Matter Volumes in the Stratified Models Y-axes represent the probability of gray matter within the significant clusters identified in SPM analyses. X-axes represent having experienced a stressful life event (0 = no, 1 = yes). (A) Unemployment among men: thresholded at uncorrected *p* < 0.001 at voxel level and family-wise error *p* < 0.05 at cluster level. Medial temporal results are shown as a binary mask overlaid into a T1 structural image. (B) Severe financial loss among women: thresholded at uncorrected *p* < 0.001 at voxel level and family-wise error *p* < 0.05 at cluster level. (C) Unemployment among those with higher education: thresholded at uncorrected *p* < 0.001 at voxel level and family-wise error *p* < 0.05 at cluster level. Medial temporal results are shown as a binary mask overlaid into a T1 structural image. All models were adjusted for age, total intracranial volume, *APOE* ε4-carrier status, history of cardiovascular disease, history of psychiatric disease, all other stressful life events, education (only panels A and B), and gender (only panel C). SLE = stressful life event.

Stratified voxel-wise analyses showed that the associations of gray matter volumes with unemployment were driven by men, whereas those with financial loss were driven by women with only partial support from interaction analyses. Thus, becoming unemployed was associated with reduced gray matter volume bilaterally in the postcentral and precentral gyrus, juxtapositional lobule cortex, superior frontal gyrus, anterior cingulate gyrus, paracingulate gyrus, hippocampus extending to the parahippocampal gyrus, thalamus, amygdala, superior temporal gyrus, and left frontal pole (Figure 3A) in men. This pattern was similar in interaction analyses, but more restricted at uncorrected 0.005 at voxel level and FWE correction at cluster level (eFigure 2). Among women, no associations were found between becoming unemployed and gray matter volumes.

Severe financial loss was associated with reduced gray matter volume bilaterally in the precuneus and posterior cingulate gyrus (Figure 3B) only among women. However, this result was not supported by a significant interaction effect. At uncorrected 0.005 at voxel level and FWE correction at cluster level, there was no interaction between gender and financial loss on gray matter volume.

### Association of Specific SLEs With Amyloid, Tau, Neurogranin, and Brain Integrity by Educational Level

In analyses stratified by educational level, death of partner was associated with a lower Aβ42/40 ratio (*p* = 0.012; eTable 12), higher p-tau (*p* = 0.003; eTable 13), and higher neurogranin (*p* = 0.011; eTable 14) only among less educated individuals. There was no interaction of death of partner with education on Aβ, p-tau, or neurogranin (eTable 11).

Finally, stratified and interaction voxel-wise analyses showed that among those with a higher educational level, becoming unemployed was associated with reduced gray matter volume bilaterally in the lingual gyrus, occipital fusiform gyrus, anterior parahippocampal gyrus, amygdala, insular cortex, anterior and posterior cingulate and paracingulate gyri, superior frontal gyrus, frontal pole, frontal orbital cortex, subcallosal cortex, frontal medial cortex, precentral and postcentral gyri, hippocampus, and parahippocampal gyrus and in the left anterior temporal fusiform cortex and left putamen (Figure 3C). This pattern was similar in interaction analyses, but restricted to the posterior areas at uncorrected 0.005 at voxel level and FWE correction at cluster level (eFigure 3). Among those with a lower educational level, no associations were found of any SLEs with gray matter volumes.

### Sensitivity Analyses

To rule out the effects of the total accumulated SLEs on the results, we repeated the analyses for death of partner, unemployment, and financial loss by adjusting for the total accumulated SLEs. We also repeated the analyses, adjusting them only for covariates and no other SLEs. The results remained unchanged in both CSF and SPM analyses (eTables 15 and 16). We further performed interaction analyses with history of psychiatric disease for the SPM and CSF main analyses that were significant and found that history of psychiatric disease interacted with death of partner on p-tau and neurogranin (eTable 17).

## Discussion

In cognitively unimpaired individuals at an increased risk of AD dementia, we found that (1) less educated participants and women showed a higher prevalence of SLEs and (2) grief-related stressors—notably death of partner—were related to AD pathophysiology while economic stressors were related to gray matter volume. Furthermore, our results suggest (3) sex- or gender-related stress pathways—involving Aβ in men and tau and neurogranin in women—and (4) an SLE-dependent influence on gray matter volumes, with unemployment showing an effect among men and those with higher education and financial loss showing an effect among women.

Our results indicate that having experienced death of partner is associated with all CSF biomarkers within the whole sample in an age-independent manner. Indeed, death of partner is considered to be one of the most stressful events in life^1^ and is associated with an increased dementia risk.^21^ However, our results are not consistent with those of a previous cross-sectional study among cognitively unimpaired participants and patients with mild cognitive impairment reporting no association between spouse bereavement and AD pathologies.^22^ Similarly, another longitudinal study, among community-dwelling cognitively unimpaired older adults, found no difference in baseline Aβ levels by widowhood status but did report widowhood to be associated with accelerated Aβ-related cognitive decline.^13^ It is possible that participants in our cohort—enriched by a family history of sporadic AD and with a higher percentage of *APOE* ε4-carriers compared to the population—are more vulnerable to stress effects on AD pathophysiology. Furthermore, our study's participants were younger and lost their partner earlier. It is also possible that unhealthy lifestyles (that could have contributed to partner's death) could explain some of the association between partner's death and higher AD pathophysiology because spouses often tend to share a similar lifestyle.^23^ Differential results could also be due to cultural differences among cohorts affecting family attachments and, therefore, bereavement effects. Although our finding is novel and warrants replication in other cohorts, it indicates that widowed individuals could be particularly susceptible to higher AD pathophysiology and as a high-risk group could possibly benefit from stress-coping interventions.

We found moderate evidence suggesting a gender-related effect of death of partner. Analyses stratified by gender indicated that the association of death of partner with Aβ might be driven by masculine gender. Men might be disproportionally affected by losing a partner because they typically have a smaller social network than women,^24^ so their loss could cut more deeply into their social network. Conversely, because women tend to have higher social support and greater social circles,^24^ this may buffer against the negative effects of losing a partner. However, we also observed that the association of death of partner with p-tau and neurogranin might be driven by feminine gender. It is possible that stress could have sex-specific and gender-specific effects on AD pathophysiology, having stronger effects on Aβ among men and stronger effects on p-tau among women. These results are in line with findings suggesting higher levels of tau pathology in women compared with men.^25^ Whether higher tau accumulation in women is linked with the prevalence and/or impact of specific risk factors, such as depression, which could confound the association between stress and AD pathophysiology, warrants further research. It is also possible that the observed moderate gender differences in CSF outcomes are due to limited statistical power as suggested by significant differences but overlapping CIs. Therefore, replication of these analyses in other cohorts is warranted. In addition, we found that death of partner is associated with higher p-tau and neurogranin only among those with lower educational level. This could be due to smaller social networks or limited financial resources and increased financial stress after the death of partner^26^ among the less educated.

We found that having experienced unemployment is associated with lower brain structure in areas of the limbic system, specifically in the anterior cingulate gyrus and paracingulate gyrus. These areas are known to be involved in emotion and behavior regulation,^27^ and lower gray matter volume of these areas is associated with neuropsychiatric symptoms in individuals with AD.^28^ Thus, our results could reflect the impact of unemployment-related stress in the limbic system. On the contrary, it is possible that the association of unemployment with lower brain structure could be explained by lower levels of cognitive stimulation.^29^ Previous research has shown an association between higher education and higher gray matter volumes in the cingulate among cognitively unimpaired individuals.^30^ It is also well documented that unemployment is associated with an increased dementia risk and has a negative impact on mental health, particularly among men.^31^

Our results suggest that the association between unemployment and lower brain structure might be driven by men and those with a higher educational level as supported by stratified and interaction analyses. Men might be more affected by unemployment and consequent financial strain because of cultural expectations of men generally being the breadwinners and stigmatization being stronger against unemployed men compared with unemployed women.^32^ Furthermore, in general, those with a lower educational level—and often fewer financial resources—already have higher levels of stress and unemployment, whereas those more highly educated are at lower risk of unemployment^9^ and the impact of unemployment may be thus greater, explaining our results.

We further found that having experienced financial loss is associated with lower gray matter volume in the frontoparietal lobe. Because these results are novel, they are difficult to compare with previous research. However, they are in line with evidence showing financial hardship to be associated with lower volumes of the amygdala and hippocampus in the general population.^33^ Our stratified analyses showed that the association of experiencing financial loss with lower brain structure could be driven by women. This could be due to sociocultural aspects that lead to inequities between men and women. Historically, women have had less access to education and the workforce, occupations have been segregated by gender, and women often face discrimination in the workplace that can lead to excess stress and reduced income.^34^ Indeed, it has been found that women stress about their finances more, affecting their mental health, compared with men.^35^ Furthermore, women, compared with men, are more likely to fill roles that are considered unpaid labor, such as caregiver roles.

Taken together, specific stressors, notably grief-related and economic-related stressors, seem to affect AD pathophysiology and brain structure, respectively. Exposure to SLEs may increase AD vulnerability through multiple mechanisms, for example, through physiologic changes, taking place in the immune, endocrine, and cardiovascular systems. Stress activates the hypothalamic-pituitary-adrenal axis releasing glucocorticoids that can cause structural brain damage.^36,37^ Furthermore, exposure to SLEs might increase unhealthy behaviors, such as smoking, which themselves are associated with an increased AD risk.^38^ In addition, exposure to stressors can increase negative affect, such as depressive symptoms.^39^ Indeed, SLEs have a substantial causal association with major depression, which is also associated with higher AD risk.^38^ The observed gender differences could be explained by differential cultural expectations and gender roles. Conversely, there are established sex differences in the stress response explained by gonadal hormone effects and sex chromosomes that could account for some of the sex and gender differences found in our study.^40,41^

The strengths of this study include the life-course evaluation of SLEs and the large sample size that allowed the stratification of analyses. Several limitations also do exist. These include the cross-sectional design, calling for future studies to investigate the change in AD pathophysiologic markers and brain structure in relation to SLEs. The possibility of reverse causality with some of the SLEs exists, for example, severe financial loss, where individuals in the AD *continuum* might be more inclined to engage with risky financial decisions. Furthermore, we cannot exclude the possibility of recall bias, especially regarding early life events. However, the events were evaluated through a structured interview translating to lower possibility of bias compared with traditional SLE checklists, and we found good test-retest correlation within our study. We did not consider the salience, duration, timing, or type of events. Future studies would benefit from examining whether the timing or age at which specific SLEs are experienced is important.^42^ Furthermore, this study focused on gender as a self-reported and self-identified binary category (i.e., woman/man), and it was presumed that all participants were cisgender. Therefore, in this study, the specific distinction between biological sex and gender was not explicitly investigated. Finally, the study sample consisted primarily of participants who identified as White, limiting the generalizability of the results to other populations. It is important to note that race and ethnicity data were not collected in a standardized manner, requiring participants to select from a combined set of race and ethnicity categories. Future research examining whether these results generalize to individuals from more diverse backgrounds is needed.

Our findings provide novel evidence suggesting that lifetime exposure to grief-related and economic-related SLEs may affect brain health. We demonstrate that the death of a partner is associated with AD pathophysiology and that unemployment and financial loss are associated with reduced gray matter volume, and that the strength of these associations depends on gender and educational level. Psychosocial interventions may represent strategies to improve stress coping among these susceptible groups identified in our study. Future research is warranted to replicate these results and to refine the risk profiles that would most benefit from these interventions, as well as to explore the optimal timing for these interventions.

## Glossary

Aβ: β-amyloid
AD: Alzheimer disease
ALFA: ALzheimer's and FAmilies
FWE: family-wise error
p-tau181: phosphorylated tau 181
SLE: stressful life event
